# The Transcriptomic Response of the Boll Weevil, *Anthonomus grandis grandis* Boheman (Coleoptera: Curculionidae), following Exposure to the Organophosphate Insecticide Malathion

**DOI:** 10.3390/insects14020197

**Published:** 2023-02-16

**Authors:** Lindsey C. Perkin, Zachary P. Cohen, Jason W. Carlson, Charles P.-C. Suh

**Affiliations:** 1 Insect Control and Cotton Disease Research Unit, Southern Plains Agricultural Research Center, Agricultural Research Service, United States Department of Agriculture, 2771 F and B Road, College Station, TX 77845, USA; 2 Center for Plant Health Science and Technology, Plant Protection and Quarantine, Animal Plant Health Inspection Service, United States Department of Agriculture, 22675 N. Moorefield Rd Bldg. 6414, Edinburg, TX 78541, USA

**Keywords:** boll weevil, malathion, RNA-seq, insecticide resistance

## Abstract

**Simple Summary:**

The boll weevil, *Anthonomus grandis grandis* Boheman (Coleoptera: Curculionidae), is a pest of commercial cotton in the Americas. Eradication programs in the United States (USA) have been very successful and have reduced boll weevil occurrence to a small region in South Texas. The programs have relied almost exclusively on the chemical insecticide malathion for over forty years to treat boll weevils in the field. Despite this heavy selection pressure, the boll weevil remains susceptible to field application rates of this insecticide. Here, we present findings from an RNA-seq experiment documenting gene expression post-exposure to field-relevant concentrations of malathion, which was used to glean information about the boll weevil’s continued susceptibility to this insecticide. Additionally, we incorporated whole genome sequence data from nearly 200 pest individuals obtained from three distinct geographical areas (Texas, Mexico, and Argentina) to determine SNP frequency in the malathion target site: acetylcholine esterase. No evidence was found from gene expression or single nucleotide polymorphism (SNP) data consistent with a mechanism of enhanced tolerance or resistance adaptation in the boll weevil, corroborating long-term field observations.

**Abstract:**

Insecticide tolerance and resistance have evolved countless times in insect systems. Molecular drivers of resistance include mutations in the insecticide target site and/or gene duplication, and increased gene expression of detoxification enzymes. The boll weevil, *Anthonomus grandis grandis* Boheman (Coleoptera: Curculionidae), is a pest of commercial cotton and has developed resistance in the field to several insecticides; however, the current organophosphate insecticide, malathion, used by USA eradication programs remains effective despite its long-term use. Here, we present findings from an RNA-seq experiment documenting gene expression post-exposure to field-relevant concentrations of malathion, which was used to provide insight on the boll weevil’s continued susceptibility to this insecticide. Additionally, we incorporated a large collection of boll weevil whole-genome resequencing data from nearly 200 individuals collected from three geographically distinct areas to determine SNP allele frequency of the malathion target site, as a proxy for directional selection in response to malathion exposure. No evidence was found in the gene expression data or SNP data consistent with a mechanism of enhanced tolerance or resistance adaptation to malathion in the boll weevil. Although this suggests continued effectiveness of malathion in the field, we identified important temporal and qualitative differences in gene expression between weevils exposed to two different concentrations of malathion. We also identified several tandem isoforms of the detoxifying esterase B1 and glutathione S-transferases, which are putatively associated with organophosphate resistance.

## 1. Introduction

The boll weevil, *Anthonomus grandis grandis* Boheman (Coleoptera: Curculionidae), was first reported in the United States (USA) in Brownsville, Texas, in 1892 and quickly spread throughout the Cotton Belt, reaching the east coast by 1916 [1]. However, concerted state and federal efforts to eradicate the boll weevil from commercial growing regions of the USA have successfully eliminated this pest, excluding a relatively small but highly productive region in the Lower Rio Grande Valley, Texas, USA (P. Burson, personal communication). Eradication in this region remains difficult due to favorable climate for both the endemic insect and malvaceae host plants, including volunteer cotton plants, which allow weevils to reproduce year-round. Eradication progress in this area is further complicated by the existence of weevil populations in Tamaulipas, Mexico, that readily disperse into the USA. In fact, a recent study found evidence of population turnover or replacement in South Texas due to seasonal recolonization [2]. In the USA, current control strategies rely on practices such as timely crop destruction, pheromone traps to monitor the presence of weevils, and insecticide applications to treat infestations [3]. Historically, different insecticides were used including organochlorines (DDT), organophosphates (parathion, chlorpyrifos, malathion), and pyrethroids (cypermethrin, pymetrozine, methomyl). However, field-evolved resistance, cost, and public pressure to limit the use of these chemicals have limited current controls in the USA to malathion [4,5,6] and the pyrethroid, Mustang Max (zeta-cypermethrin). Despite USA eradication programs’ reliance on malathion for the past forty years, no long-term tolerance or field evolved resistance to this insecticide has been reported [6]. The mechanisms that allow the boll weevil to remain susceptible to malathion are unknown but increases in weevil tolerance to malathion within a growing season have been observed [7]. However, the seasonal increase in tolerance to malathion appears to reset at the beginning of the following year; suggestive of a fitness cost for acquired tolerance. Additionally, admixture with wildtype susceptible weevils developing on untreated volunteer cotton may help dilute the allele(s) responsible for malathion resistance/tolerance. Regardless, increases in weevil tolerance to malathion does not appear to be inherited over multiple generations. Similar observations have been made for tobacco budworm and tarnished plant bug, which exhibit seasonal resistance to pyrethroids, organophosphates, carbamates, and endosulfan [4,8,9].

Malathion is an organophosphate (OP) insecticide that inhibits acetylcholinesterase (AChE) at the nerve synapse, resulting in an overaccumulation of acetylcholine, which leads to overexcitation of the nervous system and subsequent death [10]. Malathion is toxic to all animals, including humans, where it has been linked to various cancers, disruption of hormone pathways, and DNA and chromosomal damage [11,12]. Because of its toxicity to humans, malathion use is banned in many countries. Thus, most of the research or use of malathion has been limited to agriculture and mosquito control in the USA, Canada, and Sweden. In cases where OPs have been repeatedly used to control insects, many groups of insects have evolved resistance or tolerance to OP formulations, which either involve non-synonymous mutations at codons of the AChE enzyme target, inhibiting OP binding, or overexpression of Phase I detoxifying enzymes [13,14]. It has been suggested that target site insensitivity appears to be the most common mechanism of resistance in monophagous insects such as the boll weevil [4]. Non-synonymous mutations have been identified in various OP resistant insect pest systems such as the fruit fly, *Drosophila melanogaster* Meigen [15], silverleaf whitefly, *Bemisia tabaci* (Gennadius) [16], cotton aphid, *Aphis gossypii* Glover [17], olive fruit fly, *Bactrocera oleae* (Gmelin) [18], two-spotted spider mite, *Tetranychus urticae* Koch [19], new world screwworm, *Cochliomyia hominivorax* (Coquerel) [20], and the Colorado potato beetle, *Leptinotarsa decemlineata* (Say) [21,22].

Furthermore, some resistant insect systems have increased detoxifying enzyme activity such as esterases, glutathione S-transferase (GST), cytochrome P450s (CYP450s), and ATP-binding cassette (ABC) transporters due to copy number variation, recombinant forms, and/or elevated expression relative to susceptible populations [23]. For example, *Culex* mosquitoes have two esterases (gene amplification) that are overexpressed in insects tolerant to OPs compared to susceptible mosquitoes. Similarly, malathion resistance in *Anopheles* mosquitoes has been kinetically characterized as an increased expression of Phase I “B” esterases (esterase B1) [24]. GSTs are a family of enzymes that conjugate to OP insecticides, resulting in detoxification [25,26]. Recombinant forms of GSTs have been linked with OP resistance for the diamondback moth, *Plutella xylostella* (L.), and the housefly *Musca domestica* L. [27,28]. CYP450s are ubiquitous with insecticide resistance evolution and represent a large enzyme family with hyper diversity in form and function [29]. Upregulation of CYP450s has been associated with OP tolerance and resistance in the coffee leaf miner, *Leucoptera coffeella* Guérin-Méeville [30] and the fall armyworm, *Spodoptera frugiperda* (J.E. Smith) [31]. Lastly, ABC transporters have an important role in cellular detoxification, maintenance, and insecticide resistance and have been associated with OP resistance in *Aedes* and *Culex* mosquitoes [32,33].

Although resistance to malathion has not been observed at the field level, given the boll weevil’s rapid development of resistance to other OPs during early USA eradication efforts and the fact that there are no other practical alternatives, development of resistance to malathion is a major concern. In this paper, adult boll weevils were exposed to two concentrations of malathion and the transcriptomic response was monitored over a 24 h period to provide foundational knowledge on gene differential expression, which may be useful for assessing the effectiveness of alternative pesticides. In this study we focused on the genes that were differentially expressed in both treatment groups and previously associated with detoxification of insecticide, such as AChE, esterases, GSTs, CYP450s, and ABC transporters. Additionally, a large collection of boll weevil whole-genome resequencing data was used to determine SNP frequency in the malathion target site among demographically isolated populations.

## 2. Materials and Methods

### 2.1. Malathion

Technical grade malathion (American Cyanamid, Princeton, NJ, USA) was diluted in molecular grade acetone to achieve two concentrations: 1 μg/mL and 40 μg/mL. Glass 25 mL vials were coated with each malathion concentration by adding 0.5 mL of malathion solution in each vial and placing vials on a roller to allow solution to coat the inside of the vial and evaporate. Because 0.5 mL was added to each vial, the actual concentration of exposure was 0.5 and 20 μg/vial, which we refer to as the low and high rates of malathion, respectively. The 0.5 μg/vial is equivalent to the field application rate of 0.877 l/ha, which is used by the Texas Boll Weevil Eradication Foundation (TBWEF). Based on periodic tolerance testing done by the TBWEF and the Animal and Plant Health Inspection Service (APHIS), this dose has been shown in the field to kill insects within 72 h (P. Burson, personal communication; data not shown). The high rate, 20 μg/vial, was selected based on a previous malathion resistance study [7] and to ensure weevils were exposed to an adequate concentration of malathion to elicit gene responses but remain alive by the end of the study. This concentration killed 25% of weevils by the end of the 24 h experimental time point (see below) and has been shown to cause over 95% mortality by 48 h (P. Burson, personal communication; data not shown).

### 2.2. Weevil Samples

Boll weevil adults were reared from larval-infested squares collected from plants in a commercial cotton field near Edinburg, TX, USA. This field had not previously been sprayed with malathion and boll weevils were collected late season (August 2019). Squares were held at 27 ± 1 °C and 14:10 [L:D] photoperiod and were checked at least once daily for pupae. Pupae were harvested, placed in 25 mL Petri plates containing a thin layer of moistened vermiculite, and held under the same environmental conditions as infested squares. A total of 210 newly eclosed weevils were held individually in 25 mL Petri plates and fed squares daily for 3 to 4 days (held under the aforementioned conditions throughout the feeding period). Following the feeding period, six live weevils were randomly selected to serve as the control group (not exposed to malathion). The remaining weevils were randomly selected and individually placed in vials coated with either a low or high concentration of malathion as described above. Vials were capped with organza secured with rubber bands and the vials were placed on their sides in an environmental chamber to maximize weevil contact with malathion residues. The weevils were held under the same environmental conditions described above. For each concentration of malathion, 12 live weevils were randomly collected at 2, 4, 6, 8, 12, and 24 h after exposure. Malathion-treated and control weevils were placed individually in 1.5 mL microcentrifuge tubes (USA Scientific, Ocala, FL, USA) containing ~200 μL of RNAlater, and then gently crushed with a clean pestle to expose soft tissue to RNAlater. Weevil samples were stored in −20 °C until RNA extraction.

### 2.3. RNA Extraction and Sequencing

Total RNA was extracted from individual weevils using the RNeasy extraction kit (Qiagen, Hilden, Germany). Samples were checked for quantity and quality using a Tapestation 4200 (Agilent, Santa Clara, CA, USA). Total RNA from 54 samples (all six control weevils and four weevils for each combination of malathion concentration and time post-exposure) were submitted to Texas A & M AgriLife Genomics and Bioinformatics Service (TAMU TxGen, College Station, TX, USA) for purification, library preparation, and sequencing on an Illumina NovaSeq (Illumina, San Die-go, CA, USA) with a paired-end read length of 2 × 150 base pairs. Reads can be found in the NCBI SRA (BioProject ID PRJNA925810).

### 2.4. RNA-Seq Analysis

Differential gene expression analysis was performed across time within each concentration of malathion exposure using a gene-wise negative binomial generalized linear model. Significant differences were detected using the quasi-likelihood F-test as described by Chen et al. [34]. Read counts per gene were controlled for coverage and normalized within each time point, then tested for significance with a contrast design of time 0 (control), 2 h post-exposure, 4 h post-exposure, etc. until the 24 h time point. Gene ontology enrichment of DEGs by treatment was measured using the Fisher exact statistic with daughter ontology term elimination using the TopGOv2.48.0 package in R [35].

### 2.5. Acetylcholinesterase SNP Diversity across Populations

Medium coverage (2.5–7×) whole genome short read data representing three populations of *A.g. grandis* (*n* = 194) were analyzed for polymorphic differences at the AChE locus using the variant calling procedure by TAMU TxGen services described in Raszick et al. [2] (BioProject ID PRJNA623635). Biallelic polymorphisms were isolated from an imputed variant call file (vcf) at 2 MB up and downstream windows of the AChE locus. The official gene set and annotation file [36] were cross referenced for relevant genes within the AChE locus, and ideograms of genic regions were generated using the R package karyoploteR v1.22.0 [37]. In order to determine how the AChE gene locus might be evolving among pest populations, we measured Tajima’s D within the 4 MB region using a window size of 1000 bp with 250 bp steps in vcfkit v1.4 [38]. Furthermore, *D* values for each population at the AChE locus were compared against the 1% and 99% *D* values, which indicate strong selection or population expansion and balancing selection or sudden population contradiction, respectively [39].

## 3. Results and Discussion

**Figure 1:**
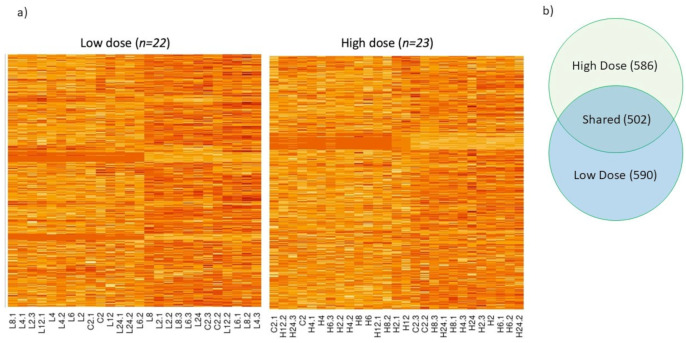
(**a**) Heat map depicting transcript abundance in the boll weevil across a 24 h time course (0, 2, 4, 6, 8, 12, and 24 h) following exposure to a low and high concentration of malathion. The color scheme indicates the gradient from high to low transcript abundance, where dark red coloring indicates the most transcripts and light yellow indicates the least while rows represent genes and columns represent individuals at time points. (**b**) Venn diagram of shared and unique genes in the low and high concentration treatment. The high concentration had a total of 1088 genes that changed significantly over the time series and 1092 in the low concentration. There were 502 genes shared between the low and high concentration.

Despite the extensive and long-term use of malathion throughout the USA Cotton Belt to eradicate the boll weevil, heritable resistance to this insecticide has not been reported in the boll weevil. To understand the biology of weevil susceptibility to malathion, an RNA-seq experiment was conducted where adult boll weevils were exposed to one of two concentrations of malathion in the laboratory. In this experiment weevils were sampled at 2, 4, 6, 8, 12, and 24 h post-exposure. RNA-seq analysis revealed 1088 and 1092 genes that significantly changed over time in boll weevils following exposure to the high and low concentrations of malathion, respectively (Figure 1a). Nearly half of these expressed genes (502 genes) were commonly shared between weevils exposed to the two different concentrations of malathion. Of the 502 genes, 286 were up regulated and 208 were downregulated in both the high and low dose treatments (Figure 1b). The remaining eight genes were also significantly differentially expressed, but patterns of expression differed between treatment groups. We focused our subsequent analyses on the 502 shared genes as these genes are likely to be biologically relevant to malathion exposure (Table S1).

### 3.1. Acetylcholinesterase Expression Is Similar after Exposure to Low and High Concentrations of Malation

**Figure 2:**
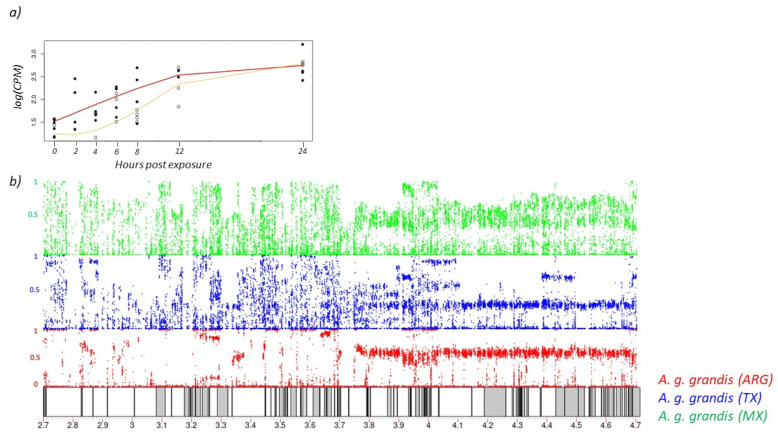
(**a**) Acetylcholinesterase (AChE) gene expression (log transformed counts per million) in the boll weevil over 24 h. The yellow line indicates the transcriptomic response to the low concentration treatment and the red line indicates the high concentration treatment. Closed circles represent gene expression values for individual weevils exposed to the high concentration of malathion, and open circles are weevils exposed to the low concentration. (**b**) Schematic of the 4 MB region on Chromosome 8 flanking the AChE gene. Gray bars indicate 88 annotated genes. The AChE gene is in the center. Colored dots represent 22,221 SNPs among the three weevil populations. *Anthonomus grandis grandis* population from Argentina is shown in red, Texas population in blue, and Mexico population in green. The *y*-axis for each of the colored sections is minor allele frequency where a frequency of one indicates fixation of the minor allele.

AChE, the OP target site, was among the 286 genes that significantly increased over time in both the high and low concentration treatments, demonstrating the relevancy of this gene in OP mode of action (Table S1). AChE transcript abundance increased over the first 12 h for both treatment groups but increased more rapidly in weevils exposed to the high concentration of malathion compared with those exposed to the low concentration (Figure 2a). However, AChE peaked in expression at 12 h for both concentrations and plateaued by 24 h. The total amount of AChE transcripts produced at 24 h was approximately double the amount at time zero for both concentrations of malathion exposure. This may indicate a physiological upper limit to the amount of AChE transcribed or a limitation due to other cellular responses from the insecticide such as apoptosis [40].

### 3.2. Population SNP Diversity and Lack of Selection at OP Target Site

AChE insensitivity to OPs, due to amino acid differences, has been described in numerous arthropod systems [15,16,17,19,22]. To visualize SNPs at the boll weevil AChE locus, approximately 200 individuals collected from geographically distinct boll weevil populations (Texas, Mexico, and Argentina) were examined. Isolation of the 4 MB region on Chromosome 8, flanking the AChE gene, yielded 88 annotated genes with 22,221 SNPs among the three weevil groups (Figure 2b). Most of the SNPs within the AChE locus reside in introns and given the relatively high Tajima *D* values, there is no evidence of directional selection within this region for any population (Figure S1). However, *D* values among these populations recapitulate prior demographic work [2] of founder events in Argentina and admixture between boll weevil populations in Mexico and Texas.

### 3.3. Expression of Other Detoxification Enzymes

In addition to target site insensitivity, insecticide resistance has been linked with metabolic enzymes such as esterases, GSTs, CYP450s, and ABC transporters [23]. Enhanced activity of these protein catalysts, such as elevated gene expression, constitutive overexpression, or gene duplication, has been observed in arthropods that have developed resistance to insecticide [24,27,28,30,31,32,33]. Here, three esterase B1s, five CYPs, two GSTs, and five ABC transporters were significantly differentially expressed over time in both malathion treatment groups.

**Figure 3:**
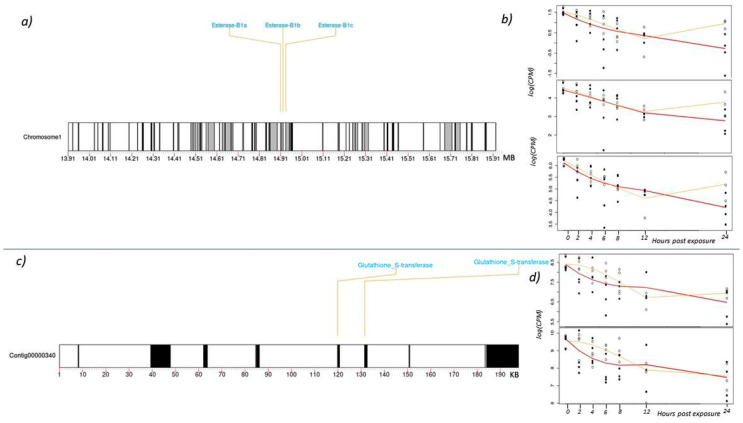
(**a**) Ideogram of the boll weevil gene region surrounding the three tandem esterase B1 genes on Chromosome 1. (**b**) Expression of each esterase B1 over 24 h (log transformed counts per million). The yellow line indicates the transcriptomic response to the low concentration treatment and the red line indicates the high concentration treatment. Closed circles represent gene expression values for individual weevils exposed to the high concentration of malathion, and open circles are weevils exposed to the low concentration. (**c**) Schematic of two tandem glutathione S-transferases (GST) and the surrounding 198 KB region on contig: ctg00000340. (**d**) Expression of each GST over 24 h (log transformed counts per million). The yellow line indicates the transcriptomic response to the low concentration treatment and the red line indicates the high concentration treatment. Closed circles represent gene expression values for individual weevils exposed to the high concentration of malathion, and open circles are weevils exposed to the low concentration.

The three esterase B1s are found in tandem on chromosome one and appear to be the result of a gene duplication event (Figure 3a). To better understand basal expression of esterase B1, a previous dataset was used to find average expression in adult boll weevils that were collected from commercial cotton fields but not recently treated with malathion [36]. That dataset showed a trend in esterase B1 expression where isoform 3 is expressed at the highest level, isoform 2 is intermediate, and isoform 1 has very low expression. The expression profiles of these esterase B1 genes after malathion exposure are also expressed at different levels at time 0, matching the trend above (Figure 3b). However, within the high concentration treatment group, all three isoforms decrease in expression over time and values are almost half of the starting value at 24 h post-exposure. In the low concentration treatment group, expression initially decreases over time but begins to increase at 12 h post-exposure and almost reaches original expression levels by 24 h. We speculate this shift and rebound in expression of esterase B1 genes is related to the cell’s ability to compensate or metabolize low concentrations of malathion. Conversely, the high concentration of malathion appears to be too toxic for cells to compensate so expression levels continue to decrease over time. Regardless, these findings emphasize the importance of routinely monitoring boll weevil populations for resistance/tolerance to malathion to ensure eradication programs are treating fields with adequate quantities of malathion.

GSTs are a large family of enzymes involved in detoxification of all major classes of insecticides as well as environmental xenobiotics and endogenous metabolites [41,42]. Glutathione conjugates to OP insecticides and results in detoxification via two distinct pathways, O-dealkylation and O-dearylation, both of which have been documented in resistant insects [25,26]. Increased GST activity via gene amplification has been observed in *M. domestica*, resulting in detoxification of OPs [43]. GST activity is also associated with upregulation of GST genes and is associated with OP resistance in *T. urticae* [44] and the red flour beetle, *Tribolium castaneum* (Herbst) [45]. In the current study, we identified two GST genes in boll weevils that decreased in expression following exposure to malathion. In the case of weevils exposed to the high concentration of malathion, both GST paralogs continued to decrease in expression throughout the entire 24 h post-exposure period. By the last post-exposure sampling period (24 h), the respective levels of expression were about a third of the initial values (Figure 3d). Similarly, expression of these two genes in weevils exposed to the low concentration of malathion also decreased but only during the initial 12 h. Thereafter, levels of expression begin to increase but did not fully recover to the initial values by the end of the last sampling period. The two GST genes were found in close proximity to one another along a 198 KB contig: ctg00000340 (Figure 3c). Upstream of the two GST genes is a Tigger transposable element (TE). TEs have been implicated as functional mutators where their insertion into a gene or gene regions can promote resistance. For example, a TE inserted in the 5′ end of a CYP450 in *D. melanogaster* was closely associated with DDT resistance [46]. Similarly, the insertion of a retrotransposon in a cadherin-superfamily gene was linked to high levels of resistance to the *Bacillus thuringiensis* (*Bt*) toxin Cry1Ac in the cotton pest *Heliothis virescens* (F.) [47]. Like many coleoptera, the boll weevil’s genome consists of nearly 60% retroelements and DNA transposons [36]. As such, mutation frequency associated with their movement might be high, leading to an increased rate in resistance evolution [48]. Thus, it is interesting that TE-mediated resistance to malathion has not materialized in the boll weevil, despite the seemingly high selection pressure and inherit potential for it to occur.

**Figure 4:**
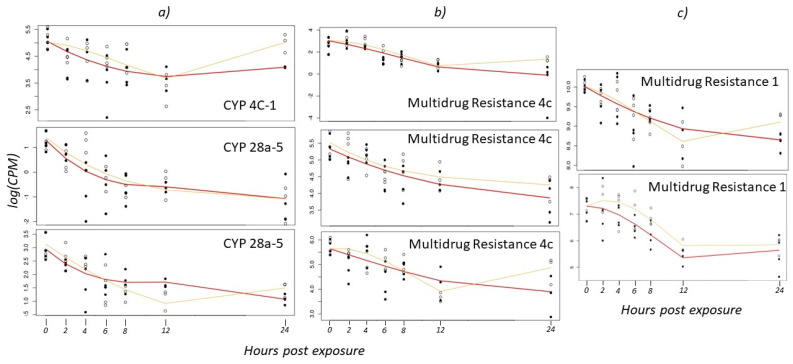
Gene expression in the boll weevil across 24 h following exposure to low and high concentrations of malathion for (**a**) three cytochrome P450 (CYP450) genes, (**b**) three multidrug resistance 4c genes, and (**c**) two multidrug resistance 1 genes (log transformed counts per million). The yellow line indicates the transcriptomic response to the low concentration treatment and the red line indicates the high concentration treatment. Closed circles represent gene expression values for individual weevils exposed to the high concentration of malathion, and open circles are weevils exposed to the low concentration.

Cytochrome P450s are a large group of xenobiotic enzymes that are induced in the cell to handle various stressors including insecticides [49]. The boll weevil has 59 CYPs in the genome annotated as xenobiotic metabolism and insecticide resistance [36], of which 23 are found in the gut [50]. In a previous study, 28 xenobiotic CYPs were shown to be constitutively upregulated in field-collected larvae and adults compared to pupae [36]. However, Brattsten [51] suggested that the lack of CYP450 inducibility in the boll weevil may be related to their susceptibility to malathion. In the current study, three CYP450 genes were significantly differentially expressed over the 24 h time course (Figure 4a). The expression profiles generally decreased over time in weevils exposed to the high concentration of malathion, which is consistent with the Brattsten [51] study. In contrast, four of the five CYPs observed in weevils exposed to the low concentration of malathion begin to increase expression at the 12 h time point, which is similar to the trend observed for the esterases and GSTs.

ABC transporters are membrane-bound proteins that mediate the efflux of compounds, including insecticides/xenobiotics and their metabolites, out of the cell [52]. The upregulation of ABC transporters in response to insecticides has been documented in an assortment of insects including mosquitoes [53,54,55], whiteflies [56], bed bugs [57], beetles [58,59], and moths [60,61]. In both *T. castaneum* and *Trichoplusia ni* Hübner, ABC transporters that are similar to multidrug resistance proteins 1 and 4 were upregulated in insects resistant to malathion [59,60]. In the current dataset, five ABC transporters were differentially expressed with one comparable to multidrug resistance proteins 1 and three analogous to multidrug resistance proteins 4 (Figure 4b,c; Table S1). These genes all show similar transcriptional trends over time, where overall gene expression decreased over the 24 h time course (Figure 4b,c). Consistent with other enzymes described above for weevils exposed to the low concentration of malathion, the 12 h time point seems to be a critical point where detoxifying enzymes begin to increase transcript abundance but do not regain initial amounts at the 24 h time point.

## 4. Conclusions

These data suggest that despite nearly forty years of extensive use of malathion in USA boll weevil eradication programs, resistance to this organophosphate has not evolved in the populations tested. We determined that both low and high concentrations of malathion elicit similar transcriptional responses in weevils with the largest concentration-response occurring within 12 h post-exposure. However, there were a few notable qualitative and temporal differences in gene expression responses between weevils exposed to the low and high concentrations of malathion. Interestingly, gene expression in weevils exposed to the low concentration of malathion used in our study, which is equivalent to the rate used in contemporary eradication programs, had a diminishing response and a quicker recovery to baseline expression with respect to the B1 esterases, ABCs, and CYPs. However, this observation does not imply weevils in South Texas have developed resistance to malathion, as the current application rate used by the TBWEF remains effective in eliminating weevils. Furthermore, SNP comparisons among three geographically distinct boll weevil populations did not indicate directional selection for AChE: the main OP target site. Therefore, there was no evidence in the SNP data of a mechanism for adaptation to malathion in the boll weevil. Molecular resistance to malathion via detoxification and target-site insensitivity has not been observed or measured in this boll weevil population. Although behavioral avoidance of malathion has been hypothesized as a mechanism of practical resistance [62], this is unlikely to account for the observed susceptibility given the history of malathion use and nearly complete eradication of the boll weevil in USA cotton-producing regions.

## Data Availability

The submission of raw sequences to NCBI SRA can be found under project number PRJNA925810.

## Supplementary Materials

The following supporting information can be downloaded at: https://www.mdpi.com/article/10.3390/insects14020197/s1, Figure S1: Tajima’s *D* values within the 4 MB region on Chromosome 8 for each of three boll weevil populations (Texas, Argentine, and Mexico). The x-axis is the Tajima’s *D* value; there was no evidence of directional selection within this region for any population but a signature of a founder event in Argentina and admixture between boll weevil populations in Mexico and Texas are noted; Table S1: List of all DE genes.
