# Supplementary material for: The Transcriptomic Response of the Boll Weevil, Anthonomus grandis grandis Boheman (Coleoptera: Curculionidae), following Exposure to the Organophosphate Insecticide Malathion
Source: Insects. 2023 Feb 16;14(2):197. doi: 10.3390/insects14020197 (PMC9960965; doi:10.3390/insects14020197)
Supplement: Supplementary file 1 [file insects-14-00197-s001.zip › insects-2130520-Supplementary Figure S1.pdf]

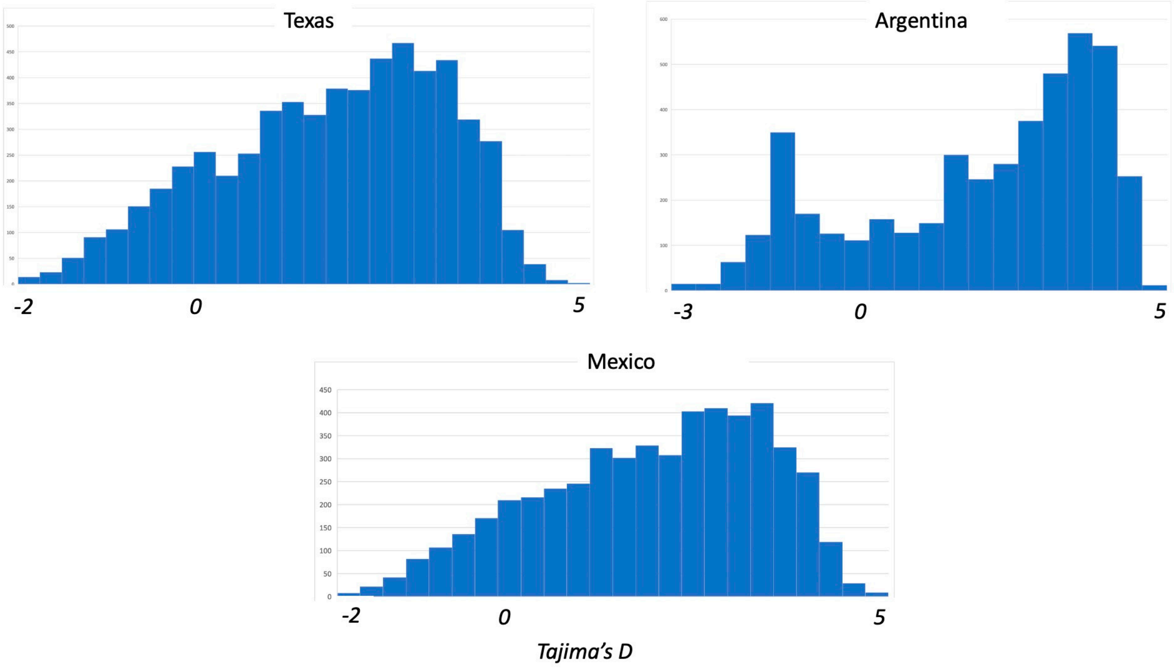

Figure S1. Tajima's D values within the 4 MB region on Chromosome 8 for each of three boll weevil populations (Texas, Argentine, and Mexico). The x-axis is the Tajima's D value; there was no evidence of directional selection within this region for any population but a signature of a founder event in Argentina and admixture between boll weevil populations in Mexico and Texas are noted.
